# Evaluating individual and mixed arbuscular mycorrhizal fungi for controlling *Rhizoctonia* root rot in lupine

**DOI:** 10.1038/s41598-025-20631-4

**Published:** 2025-10-07

**Authors:** Marwa A. M. Atwa, Sozan E. El-Abeid, Salama A. S. El-Blasy

**Affiliations:** 1 Legume and Forage Diseases Research Department, Plant Pathology Research Institute, ARC, Giza, Egypt; 2 Mycology and Plant Diseases Survey Department, Plant Pathology Research Institute, ARC, Giza, Egypt

**Keywords:** Arbuscular mycorrhizal fungi, Lupine, *Rhizoctonia solani*, Biological control, Microbiology, Plant sciences

## Abstract

Lupin is an economically and ecologically important legume crop. However, it is susceptible to infection with *Rhizoctonia solani*, which causes damping off and root rot diseases. Arbuscular mycorrhizal fungi (AMF) as a biological control agent has emerged as a promising alternative to chemical fungicides. Four species of AMF, namely *Entrophospora etunicata*, *Rhizophagus clarus*, *Rhizophagus intraradices*, *Entrophospora lutea* and their mixture were evaluated to determine their compatibility with lupine plants, also as a biocontrol agent against damping-off and root-rot diseases in comparison with the chemical fungicide Rizolex-T. All mycorrhizal treatments significantly reduced damping-off disease and increased the surviving plants under greenhouse and field conditions. The most effective isolates were *Entrophospora lutea*, followed by *R. intraradices.* Alongside their biocontrol activity, they positively enhance the uptake of macro- and micronutrients, promoting nodulation, and boosting nitrogenase enzyme activity. Additionally, they improved various plant growth parameters, increased yield, and stimulated the activity of peroxidase (PO), polyphenol oxidase (PPO), and elevated phenolic compounds. Moreover, greater accumulations of proline, chlorophyll, and carotenoids were observed. However, *Entrophospora lutea* treatment was effective as Rizolex-T in disease reduction and superior in enhancing plant growth and yield.

## Introduction

Legume crops such as faba bean, chickpea, lupine, soybean, and lentil are critical components of sustainable agriculture, providing food, feed, and ecosystem services^1^. Legumes fix atmospheric nitrogen and leave the residues of legumes in the soil after cultivation increases the productivity of the following crops^2^. Furthermore, they decreased the amount of energy needed, the potential for global warming, and the formation of the ozone layer^3^. Legume crops are very important as growing crops or as a residue^1^. Lupine (*Lupinus* spp.) is an economically and ecologically important legume crop regarded for its high-quality protein content (up to 40%), which makes it suitable for sustainable production and consumer acceptability^4^.

Despite its global importance, lupine cultivation faces region-specific challenges. In Egypt, for example, one of the most serious constraints is root rot disease caused by *Rhizoctonia solani* Kühn resulting in a considerable annual loss in crop yield^5^. The fungus is extremely challenging to control due to its very wide host range, and it can live saprophytically on living or dead plant material, or as sclerotia in the soil for more than 3 years^6^.

The fungicide Rizolex-T is widely used as both a preventive and curative agent against *Rhizoctonia* diseases^7^. Rizolex-T is composed of tolclofos-methyl and thiram. Tolclofos-methyl is classified in the Fungicide Resistance Action Committee code list (FRAC code 14) and interferes with lipid synthesis and membrane integrity, in addition to effectively inhibiting both mycelia and sclerotia development, while thiram is a multi-site contact activity (FRAC code M3)^8^.

Even though fungicides are effective in controlling the fungus, they are extremely harmful to humans, cause environmental pollution, and promote the development of resistant strains of pathogens^9^. As a result, there is an increase in environmental consciousness for the gradual switch from conventional agriculture to sustainable agricultural cropping systems that depend on biological processes rather than agrochemical treatment such as fungicides or fertilizers to preserve crop health and productivity^10^.

The use of AMF enhances nutrient availability, improves plant growth, and aids soil fertility^11^. AMF is categorized as a bio-stimulant product in the European Union, together with plant growth-promoting rhizobacteria (PGPR) and plant growth-promoting fungi (PGPF). In addition to its bio-stimulant abilities, AMF can trigger defense mechanisms that result in “mycorrhiza-induced resistance”^12^.

Recently, the application of AMF as a biocontrol strategy to control soil-borne pathogens has gained growing importance in decreasing the severity of various plant diseases and increases plant resistance to biotic and abiotic challenges^13^. They stimulate defense mechanisms and produce various metabolites that can restrict the pathogens development^14^ and induce plant systemic resistance^15^. AMF can also interact with other beneficial bacteria in the soil, increasing their biocontrol function^14^. The multifaceted approach to disease management highlights the importance of mycorrhizal fungi in sustainable agriculture^16^.

Most legumes can form mycorrhizal symbioses with AMF, thereby benefiting natural and agricultural ecosystems^17^. However, earlier studies reported that many *Lupinus* species are traditionally non-mycorrhizal or weakly colonized, which is exceptional for the family Fabaceae^18,19^. Subsequent research has challenged this view; Snyder^20^ demonstrated that *Lupinus albus* could be colonized by *Glomus fasciculatum* to a degree comparable to other cover crops, while Giovannetti et al.^21^ found that root exudates of *L. albus* did not inhibit the hyphal growth of *G. mosseae*. Moreover, recent reviews indicate that under certain environmental conditions, and depending on both plant species and AMF taxa, Lupinus species can establish mycorrhizal associations. However, AMF can make non-symbiotic interactions with lupine plants^17^.

The aim of this study was to evaluate the compatibility of selected arbuscular mycorrhizal fungi (AMF) species with lupine plants to reduce damping-off and root rot diseases of lupine. In addition, we investigated their influence on enhancing lupine plant growth and yield under greenhouse and field conditions.

## Results

### Morphological and molecular identification of the pathogen

**Fig. 1:**
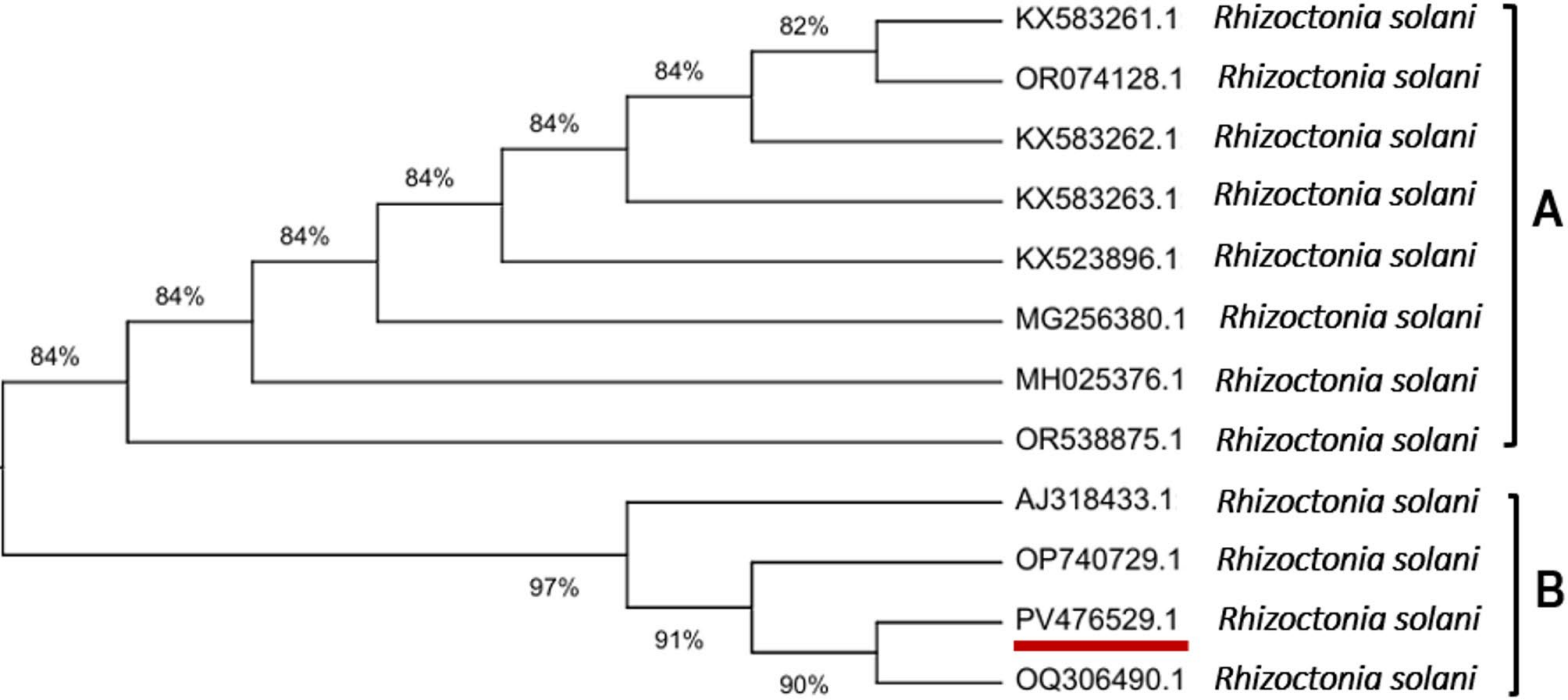
Phylogenetic tree based upon CLUSTAL W alignment of the ITS region of rDNA nucleotide sequences of 12 *Rhizoctonia solani* isolates. Maximum likelihood was used, with bootstrap values after 1000 replications of calculated runs by using MEGA11 software. The branch numbers indicate bootstrap values. Our own sequenced *R. solani* isolate (ITS: PV476529.1) is underlined with red color, and the tree shows its identity with the most similar *R. solani*, GenBank accession numbers.

According to the microscopic and morphological features of the pathogen, the isolate was identified as *Rhizoctonia solani*. Blast analysis revealed that the ITS sequence of the isolate is *R. solani* with accession number PV476529.1 (Fig. 1).

### Greenhouse experiments

#### Impact of mycorrhizal treatments and Rizolex-T on the occurrence of damping-off disease of lupine plants grown in artificially infested soil by *R. solani*

Generally, all mycorrhizal treatments led to a decrease in the percentage of damping-off disease (pre- and post-emergence) and an increase in the percentage of surviving plants (Table 1). *Entrophospora lutea* and Rizolex-T treatments have highly reduced the pre-emergence damping- off of *R. solani* on germinated lupine seeds, as there were 4% dead seeds in comparison with 40% for the infected control and 0% for the healthy control grown in non-infested soil. Such curative effect extends to the results of survived plants as 88% for both of them, followed by *Rhizophagus intraradices* and *R. clarus.* Furthermore, mycorrhizal treatments caused a reduction in the incidence of root rot disease of lupine plants; the maximum decrease was related to Rizolex-T treatment (23.1%), followed by *Entrophospora lutea* (24.3%), *R. intraradices* (30.1%), and *R. clarus* (32.2%), compared with 63.6% for infected control plants.

**Table 1:** Impact of individual and mixed arbuscular mycorrhizal fungi and Rizolex-T treatments on the occurrence of damping-off disease of lupine plants grown in artificially infested soil by *R. solani*.

Treatments	Damping-off				Survived plants %	Increasing over-infected control %	Disease index
	Pre-emergence		Post-emergence				
	Incidence %	Reduction %	Incidence %	Reduction %			
*Entrophospora etunicata*	8 c*	80	12 a	0.0	80 c	66.7	40.2
*Rhizophagus clarus*	8 c	80	8 b	33.3	84 bc	75.0	32.2
*Rhizophagus intraradices*	8 c	80	8 b	33.3	84 bc	75.0	30.1
*Entrophospora lutea*	4 d	90	8 b	33.3	88 b	83.3	24.3
Mixture	20 b	50	12 a	0.0	68 d	41.7	45.6
Rizolex –T	4 d	90	8 b	33.3	88 b	83.3	23.1
Control (*R. solani*)	40 a	0.0	12 a	0.0	48 e	0.0	63.6
Healthy control (non-infested soil)	0.0 e		0.0 c		100 a		0.0

*Means in each column followed by the same letter are not significantly different according to Duncan’s multiple range test.

(*P* ≤ 0.05).

#### Impact of mycorrhizal treatments and their mixture and Rizolex-T on some growth parameters of lupine plants grown in artificially infested soil by ***R. solani***

**Fig. 2:**
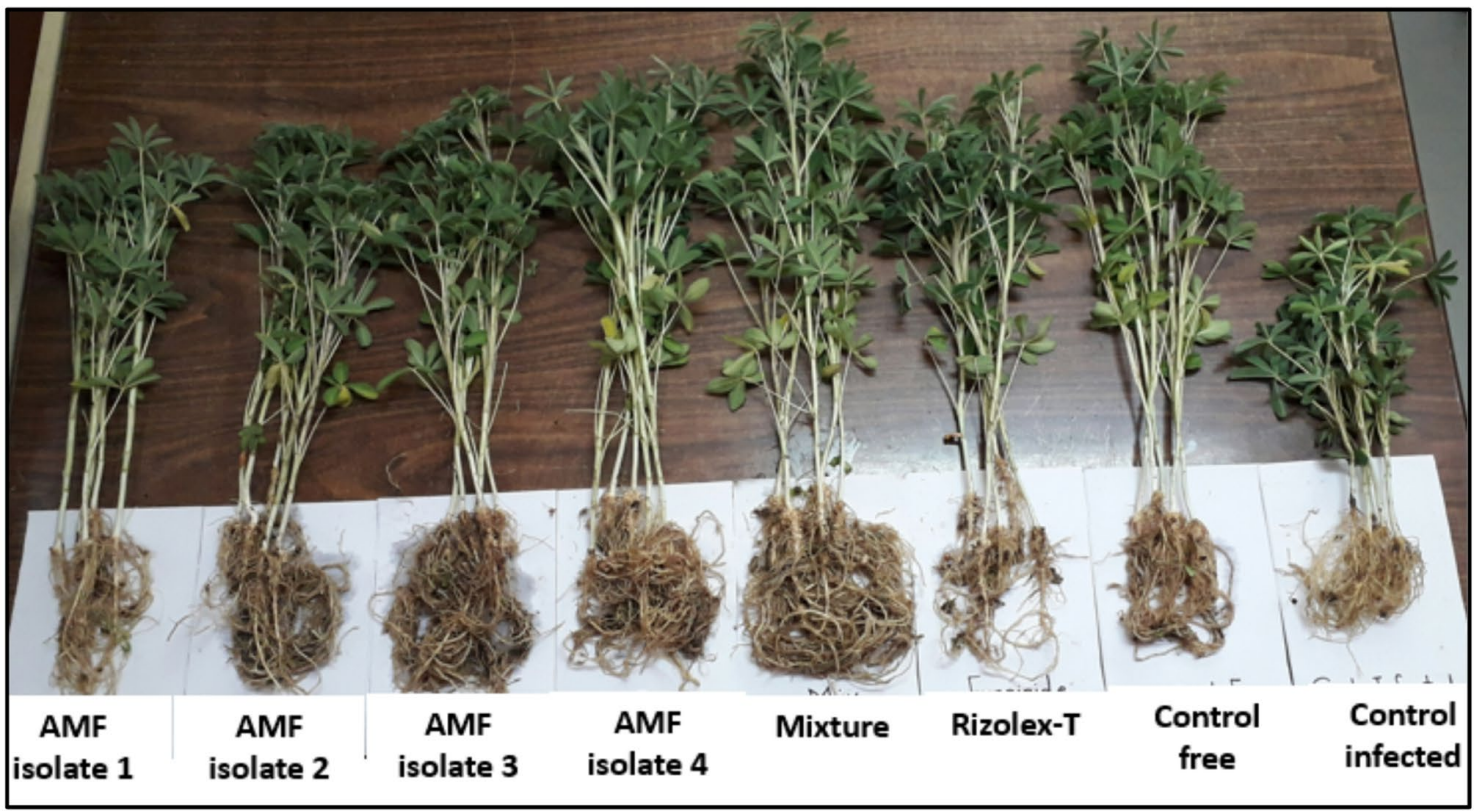
Effect of individual and mixed AMF and Rizolex-T treatments on lupine plants grown in artificially infested soil by *R. solani* after 60 days of planting. Treatments from the left: AMF isolate 1 (*Entrophospora etunicata*), AMF isolate 2 (*Rhizophagus clarus*), AMF isolate 3 (*Rhizophagus intraradices*), AMF isolate 4 (*Entrophospora lutea*), mixture of AMF isolates, Rizolex-T, Control free (non-infested soil), and Control infected (infested soil).

All mycorrhizal treatments showed stimulatory effects on plant height, fresh and dry weight of shoots and roots, as well as nodulation and nodule dry weight. On the other hand, all growth parameters were significantly reduced in lupine plants infected with *R. solani* (Table 2; Fig. 2). There is an increase in plant height with all treatments and their mixture; the maximum increase was related to the mixture treatment and *Entrophospora lutea* without any significant differences, followed by the *R. intraradices* treatment and healthy control grown in non-infested soil. The same results were also obtained for the fresh and dry weight of the shoot and root. The significant maximum increases were related to the mixture treatment, *R. intraradices*, and *Entrophospora lutea*. The results also showed that increased nodulation led to higher nitrogenase activity, and the maximum increase was related to *Entrophospora lutea*, followed by *R. intraradices* treatments. Nodulation was poor in infected control plants and Rizolex-T treatments without any significant differences; also, the activity of the nitrogenase enzyme decreased. Among mycorrhizal treatments, the lowest value of nodule number, nodule dry weight, and nitrogenase activity was related to the mixture treatment and *Entrophospora etunicata*. On the other hand, the results showed that the mixture treatment and *Entrophospora lutea* treatment exhibited a higher colonization rate in the roots at 56% and 53%, respectively, followed by *R. intraradices* and *R. clarus* at 51% and 47%, respectively. The lowest colonization rate was related to *Entrophospora etunicata* at 9%.

**Table 2:** Impact of individual and mixed arbuscular mycorrhizal fungi and Rizolex-T treatments on some growth parameters of lupine plants grown in artificially infested soil by *R. solani* after 60 days of planting.

Treatments	Plant height cm	Shoot fresh weight g/plant	Shoot dry weight g/plant	Root fresh weight g/plant	Root dry weight g/plant	Nodule number/plant	Nodules dry weight mg/plant	N2-ase activity*	AMF colonization rate %
*Entrophospora etunicata*	33 c^**^	8.5 b	2.40 c	6.85 b	2.11 b	22 d	167.98 cd	12.4	9.0 d
*Rhizophagus clarus*	35 c	11.8 a	3.04 b	6.60 b	2.16 b	26 c	182.48 c	18.6	47.3 c
*Rhizophagus intraradices*	38 b	12.5 a	3.38 a	9.47 a	3.38 a	30 b	304.25 b	22.3	51.0 b
*Entrophospora lutea*	40 ab	12.8 a	3.34 a	9.33 a	3.33 a	41 a	387.98 a	30.1	55.0 a
Mixture	42 a	12.9 a	3.45 a	9.69 a	3.48 a	19 e	159.35 d	10.4	56.0 a
Rizolex –T	35 c	6.7 c	2.20 c	3.40 d	1.50 c	12 f	99.65 e	4.1	--
Control (*R. solani*)	20 d	5.1 d	1.19 d	3.63 d	0.99 d	10 g	83.13 e	4.0	--
Healthy control (non-infested soil)	38 b	7.5 bc	2.30 c	5.72 c	1.44 c	20 e	161.45 d	11.1	--

*Nitrogenase activity µmole C_2_H_4_ /g dry nodule /h.

**Means in each column followed by the same letter are not significantly different according to Duncan’s multiple range test, (*P* ≤ 0.05).

#### Impact of mycorrhizal treatments and Rizolex-T on the contents of macro and micro elements of chickpea plants grown in artificially infested soil by *R. solani*

Data in Table 3 shows the influence of inoculation of AMF on the content of macro- and micronutrients of lupine plants. Results revealed that *Entrophospora lutea* treatment recorded the highest value of macro (nitrogen, phosphorus, and potassium) and micro (iron, manganese, zinc, and copper) nutrient contents, followed by *R. intraradices* treatment in comparison to the infected control plants. Among mycorrhizal treatments, mixture treatment recorded the lowest values as well as Rizolex-T treatment. The other treatments caused an increase over the infected control. All mycorrhizal treatments exhibited higher values of macro- and micronutrient contents over the healthy control grown in non-infested soil.

**Table 3:** Impact of individual and mixed arbuscular mycorrhizal fungi and Rizolex-T treatments on the contents of macro and micro elements of lupine plants grown in artificially infested soil by *R. solani*.

Treatments	Macro elements (mg/g dry weight)			Micro elements (mg/g dry weight)			
	*N*	*P*	K	Fe	Mn	Zn	Cu
*Entrophospora etunicata*	4.0	0.639	2.94	0.348	0.260	0.029	0.014
*Rhizophagus clarus*	4.3	0.701	3.20	0.460	0.274	0.032	0.014
*Rhizophagus intraradices*	4.9	0.792	3.51	0.290	0.290	0.035	0.015
*Entrophospora lutea*	5.3	0.871	3.93	0.531	0.320	0.040	0.015
Mixture	3.0	0.543	2.0	0.189	0.120	0.020	0.005
Rizolex –T	2.5	0.325	2.13	0.194	0.173	0.024	0.006
Control (*R. solani*)	2.0	0.209	1.94	0.178	0.103	0.017	0.005
Healthy control—(non-infested soil)	2.9	0.428	2.28	0.240	0.162	0.020	0.006

#### Impact of the mycorrhizal treatments on the activity of peroxidase, polyphenol oxidase enzymes, phenolic content, proline content and photosynthetic pigments (chlorophyll and carotenoids) contents

Data in Table 4 revealed that the activities of peroxidase (PO) and polyphenol oxidase (PPO) were significantly increased by the infection with *R. solani*. Additionally, the enzyme activity of plants treated with AM fungi was significantly higher. Similarly, the phenol content was significantly higher in the infected control than that of healthy plants (untreated). The highest activities of peroxidase were recorded in plants treated with *Entrophospora lutea*, followed by *R. intraradices* and infected control, while for polyphenol oxidase, the highest activities were related to infected control treatment. Among the mycorrhizal treatments, *Entrophospora lutea* and *R. intraradices* showed high activity, followed by *R. clarus.* Additionally, all treatments enhanced the phenolic contents over the infected control; the maximum increase was recorded in plants treated with *Entrophospora lutea* and *R. intraradices*, followed by *R. clarus* treatments. Healthy control grown in non-infested soil recorded the lowest value among all treatments regarding peroxidase, polyphenol oxidase, and phenolic contents.

Results showed that mycorrhizal isolates significantly increased the contents of proline. Significantly higher proline contents were observed with *Entrophospora lutea* treatment, followed by *R. clarus* and *R. intraradices* treatments. Meanwhile, the lowest concentrations were recorded in *Entrophospora etunicata* and the mixture treatment in comparison with infected plants. On the other hand, all mycorrhizal treatments and their mixture increased the chlorophyll levels. Among treatments, plants treated with *Entrophospora lutea* exhibited the highest amount of total chlorophyll, followed by *R. intraradices*, compared with the infected control, which recorded the lowest chlorophyll content. Additionally, *Entrophospora lutea* recorded the highest carotenoid amount, followed by *R. intraradices* and *R. clarus* treatments.

**Table 4:** Impact of individual and mixed arbuscular mycorrhizal fungi treatments on the activity of oxidative enzymes, phenolic contents, proline, and pigment contents of lupine plants grown in artificially infested soil by *R. solani*.

Treatments	Peroxidase activity absorbance at 430 nm Unit/ mg protein /min	Polyphenol oxidase activity absorbance at 495 nm Unit/ mg protein/ min	Phenolic contents catechol equivalent mg/g fresh weight	Proline mg/g/h/fresh weight	*Photosynthetic pigments mg/g fresh weight			
					Chl a	Chl b	Total Chl	CARs
*Entrophospora etunicata*	0.305 d**	0.033 d	1.021 bc	0.17 b	0.28	0.49	0.77	0.05
*Rhizophagus clarus*	0.414 c	0.056 c	1.559 ab	0.40 b	0.41	0.42	0.83	0.07
*Rhizophagus intraradices*	0.584 b	0.065 bc	1.613 a	0.32 b	0.51	0.42	0.90	0.13
*Entrophospora lutea*	0.982 a	0.070 b	1.723 a	1.28 a	0.63	0.32	0.95	0.16
Mixture	0.279 de	0.034 d	0.987 c	0.11 b	0.31	0.29	0.60	0.05
Control (*R. solani*)	0.506 bc	0.133 a	0.836 c	0.10 b	0.09	0.09	0.18	0.01
Healthy control- (non-infested soil)	0.203 e	0.028 d	0.621 c	0.38 b	0.49	0.49	0.77	0.14

*Photosynthetic pigments; chlorophyll a (Chl a), chlorophyll b (Chl b), and carotenoids (CARs).

**Means in each column followed by the same letter are not significantly different according to Duncan’s multiple range test, (*P* ≤ 0.05)

### Field experiments

#### Impact of mycorrhizal treatments and Rizolex-T on the occurrence of damping-off disease of lupine plants grown under natural infection

All AMFs significantly reduced the incidence of pre- and post-emergence damping off and increased the percentage of survived plants compared with the untreated control (Table 5). Additionally, the highest increase in survived plants over untreated control was observed with *Entrophospora lutea* followed by *R. intraradices* treatments (91.5% and 87.5%) in the first season and (92.1% and 87.8%) in the second season compared with Rizolex-T treatment (92.2% and 91.5%) respectively in the two seasons.

**Table 5:** Impact of individual and mixed arbuscular mycorrhizal fungi and Rizolex-T treatments on the occurrence of damping-off disease of lupine plants under natural infection in the 2021 and 2022 growing seasons.

Treatments	Damping- off				Survived plants %	Increasing over infected control %
	Pre-emergence		Post-emergence			
	Incidence %	Reduction %	Incidence %	Reduction %		
(A): first season 2021						
*Entrophospora etunicata*	13.5 b	48.5	2.7 bcd	89.7	83.8 cd	74.6
*Rhizophagus clarus*	12.0 b	54.2	2.7 bcd	89.7	85.3 c	77.7
*Rhizophagus intraradices*	7.7 c	70.7	4.8 bc	81.3	87.5 b	82.3
*Entrophospora lutea*	5.8 c	77.7	2.3 cd	91.0	91.9 a	91.5
Mixture	11.8 b	54.8	5.8 b	77.4	82.4 d	71.7
Rizolex –T	6.3 c	75.8	1.5 d	94.2	92.2 a	92.1
Control	26.2 a	0.0	25.8 a	0.0	48.0 e	0.0
(B) Second season 2022						
*Entrophospora etunicata*	14.5 b*	45.9	3.5 bc	85.3	82.0 c	65.9
*Rhizophagus clarus*	12.7 bc	52.9	4.3 bc	81.8	83.0 c	68.0
*Rhizophagus intraradices*	9.2 cd	65.9	3.0 bc	87.4	87.8 b	77.7
*Entrophospora lutea*	6.2 d	77.1	1.8 c	92.3	92.0 a	86.2
Mixture	12.5 bc	53.5	5.8 b	75.5	81.7 c	65.4
Rizolex –T	6.0 d	77.7	2.5 bc	89.5	91.5 a	85.2
Control	26.8 a	0.0	23.8 a	0.0	49.4 d	0.00

*Means in each column followed by the same letter are not significantly different according to Duncan’s multiple range test, (*P* ≤ 0.05)

#### Impact of mycorrhizal treatments and Rizolex-T on some growth parameters of lupine plants grown under natural infection

All AMFs treatments improved plant growth characteristics in the field, which led to an increase in lupine plant production when compared to the untreated control treatment (Table 6). Plants colonized with *Entrophospora lutea* significantly showed a greater increase in all crop parameters, followed by *R. intraradices*, compared with other mycorrhizal treatments in the two seasons. Other treatments significantly increased plant height, number of branches, number of pods per plant, seed weight per plant, and the weight of one hundred seeds more than the untreated control. The maximum figures of yield were recorded with the *Entrophospora lutea* treatment, followed by the Rizolex-T treatment and then the *R. intraradices* treatment. Mixture treatment recorded the lowest value among other treatments.

In summary, mycorrhizae *Enterophspora lutea* treatment is effective in reducing the disease without significant differences from the fungicide Rizolex-T treatment, which is thought to be one of the best mycorrhizal treatments for reducing the incidence and severity of *R. solani*. Furthermore, *Enterophspora lutea* increased the yield of lupine plants more than the Rizolex-T treatment.

**Table 6:** Impact of individual and mixed arbuscular mycorrhizal fungi and Rizolex-T treatments on growth parameters and yield under natural infection in the 2021 and 2022 growing seasons.

Treatments	Plant height (cm)	Number of branches/plants	Number of pods/ plant	Seed weight (g)/Plant	100-seed weight (g)	Seed yield (ton/fed)
(A): first season 2021						
*Entrophospora etunicata*	119.8 c*	3.5 b	26.0 c	29.35 cd	31.9 c	1.475 de
*Rhizophagus clarus*	120.8 bc	3.5 b	24.8 cd	30.7 c	32.3 c	1.572 d
*Rhizophagus intraradices*	123.8 ab	4.1 ab	32.3 b	35.2 b	38.2 b	1.846 c
*Entrophospora lutea*	128.3 a	4.9 a	37.3 a	45.0 a	46.6 a	2.377 a
Mixture	119.8 c	3.3 bc	23.5 d	27.48 d	29.9 d	1.396 e
Rizolex –T	119.8 c	4.0 ab	32.3 b	36.7 b	38.0 b	2.073 b
Control	90.8 d	2.5 c	17.8 e	21.6 e	26.4 e	0.822 f
(B): Second season 2022						
*Entrophospora etunicata*	121.8 cd	3.4 cd	27.5 c	29.3 cd	32.5 d	1.448 e
*Rhizophagus clarus*	123.3 bc	3.8 bc	27.5 c	31.9 c	33.6 d	1.592 d
*Rhizophagus intraradices*	126.8 b	4.3 ab	33.8 b	36.2 b	39.8 c	1.878 c
*Entrophospora lutea*	131.3 a	4.8 a	38.0 a	45.1 a	45.5 a	2.298 a
Mixture	118.8 d	3.3 cd	27.0 c	27.7 d	29.3 e	1.382 e
Rizolex –T	120.0 cd	3.8 bc	34.5 ab	37.0 b	41.6 b	2.078 b
Control	92.8 e	2.8 d	17.5 d	21.8 e	25.8 f	0.831 f

*Means in each column followed by the same letter are not significantly different according to Duncan’s multiple range test, (*P* ≤ 0.05).

## Discussion

In literature, Lupinus is considered a nonmycorrhizal or weak mycorrhizal genus, which is exceptional in Leguminosae^18^. However, mycorrhizal colonization by genus *Glomus* was observed in some of the Lupinus species, which indicated that the root exudates of *Lupinus albus* did not inhibit the hyphal growth of *G. mosseae*^21,22^. A more recent review pointed out that mycorrhizal fungi can colonize *Lupinus* species at low rates depending on lupine species, fungal taxa, and growth condition^23^.

In the present study, results indicated that all mycorrhizal isolates were effective in decreasing pre- and post- emergence damping off as well as increasing the surviving plants by various degrees. *Enterophspora lutea* treatment was successful in reducing the disease without any significant differences with the fungicide under greenhouse and field conditions, followed by *R.intraradices* and *R. clarus.*

However, the AMF are considered as ideal biocontrol agents due to their ability to establish symbiosis relationships with the roots of more than 90% of plants^17^. Moreover, *Glomus* species could be an important tool to control some soil-borne pathogens by pre-activating the plant defense response, increasing plant nutrient absorption and increasing resistance to abiotic stresses^24^.Several studies revealed that *Glomus* spp. are potential biocontrol agents against damping-off disease caused by *R. solani*^25^. Additionally, inoculated plants with mycorrhizal fungus *G. mosseae* showed a lower disease severity of *R. solani* than *G. clarum*^24^.

The efficacy of mycorrhizal treatments in reducing disease severity may be attributed to improving nutrient status in the rhizosphere, which reduces direct competition for space and resources with pathogens. It also allows host plants to be more vigorous and, consequently, more resistant or tolerant to pathogen attacks^26^; induces plant immunity through systemic acquired resistance (SAR) and cell wall defenses; and enhances the production of defense compounds like phenolics, *β*-1,3-glucanase, and chitinolytic enzymes^27^.

The present study concluded that the percentages of lupine root colonization by AM fungi varied with the application of different mycorrhizal species. Meanwhile, the most frequently documented response to AM colonization is the improvement of host plant nutrition, which in turn enhances plant vigor and resistance to pathogen invasion^28^. Root colonization by AMF is also considered one of the most critical indicators of symbiotic efficiency and functional compatibility in legume species^29^. Furthermore, the symbiotic relationship between mycorrhizal fungi and Lupinus species depends on the lupine species, fungal taxa, and edaphic growth conditions, and may therefore result in either positive or negative outcomes^17^. However, in some cases, exudates from lupine roots can inhibit the germ tube growth of AM fungal spores^30^. In contrast, Gianinazzi-Pearson et al.^31^ reported that lupine root exudates may instead promote spore germination and early hyphal development of a vesicular–arbuscular mycorrhizal fungus.

Also, our results demonstrate that all mycorrhizal treatments increased the contents of macro- and micronutrients during the greenhouse experiment. The maximum increase was related to *Enterophspora lutea* treatment. In this respect, AMFs have a unique and beneficial relationship with plants, as they absorb carbon generated during photosynthesis for their growth. However, plants compensate for the carbon losses by increasing photosynthesis rate, improving nutrient supply, stimulating plant growth, and enhancing crop yield^32,33^. Furthermore, AMF can colonize various plants, including legumes, and form a network of hyphae that penetrates deep into the soil around plant roots, improving nutrient intake^34^. Additionally, Jia et al.^35^ noted that increased phosphorus uptake contributes significantly to increased resistance of mycorrhizal plants to pathogens. Watts-Williams et al.^36^ also found that sorghum plants (*Sorghum bicolor*) grown on phosphate-poor soils and inoculated with *R. irregularis* generated greater yields and grains that were more nutritionally rich in phosphate, iron, and zinc.

A relational trend for the effect of mycorrhizal treatments on suppression of damping-off disease in lupine was an enhancement in the activities of peroxidase and polyphenol oxidase enzymes. Consequently, the total phenolic content in these treated plants increased. Here again, AMF secretes Microbe-Associated Molecular Patterns (MAMPs) that activate a local immune response in plant roots, known as MAMP-Triggered Immunity (MTI), and produce salicylic acid, which generates long-distance signals for systemic defense and primes defenses similar to systemic acquired resistance (SAR), including stimulation of polyphenol oxidase, *β*-1,3 glucanases, peroxidase, and phenylalanine ammonia lyase activities, as well as accumulation of phenolic compounds^12^.

Accordingly, phenolic compounds are antimicrobial and involved in the biosynthesis of lignin, which acts as a physical barrier against disease development and deposits pectin and callose around the sites of pathogen infection^37^. Moreover, polyphenol oxidase (PPO) is involved in the oxidation of ortho-diphenolic compounds into o-quinones and lignifies cell walls during microbial invasion^38^. Additionally, peroxidases (PO) participate in producing phytoalexins and reactive oxygen species (ROS) with antifungal properties that help in preventing disease development^39^.

Likewise, the results indicated that inoculation with mycorrhizal species enhanced growth parameters, nodulation, and nitrogenase enzyme activity, particularly with *Entrophospora etunicata* and *R. intraradices*. Application of AMF accelerates the production of plant growth-promoting hormones such as indole acetic acid, which improve plant growth^40^. The association of *Glomus* species with legumes significantly promotes root and shoot growth and dry weight, leading to improved nodulation and nitrogen fixation^41^. A strong synergistic interaction between AMF and rhizobia significantly enhanced nitrogen content, the number and weight of nodules in legume plants compared to uninoculated controls^42^.

On the other hand, among the mycorrhizal treatments, the lowest values of nodule number, nodule dry weight, and nitrogenase activity were recorded in the mixed inoculation treatment. In general, many plant species exhibit enhanced growth when inoculated with more than two AM fungal species; however, certain combinations can reduce host plant growth and yield. For instance, two Glomus species with similar nutrient acquisition functions might negatively affect each other^43^, thereby reducing the overall mycorrhizal benefit to the host^44^. Similarly, Shi et al.^17^ reported negative mycorrhizal dependencies for fresh and dry nodule weight of *L. latifolius* when inoculated with mixed AM fungal species (*G. intraradices*, *G. etunicatum*, and *G. intraradices*), along with decreased nutrient uptake following inoculation with *G. mosseae* and *Gigaspora margarita*. Furthermore, antagonistic AM fungal species normally grow in disturbed, open habitats and often fail to establish in closed communities^45^.

Additionally, the inoculation with *Entrophospora etunicata* and *R. intraradices* improved the growth and production of lupine plants under field conditions. However, Qiao et al.^46^ found that AMF inoculation of faba beans increased the weights of pods at harvest compared with uninoculated plants.

Results of the recent study showed that inoculating lupine with the studied mycorrhizal species improved various biochemical properties. There was a marked increase in chlorophyll, carotenoids, and proline contents. In this respect, AMF increases chlorophyll content by promoting leaf photosynthetic activity and accelerating metabolic processes; reduced chlorophyllase activity along with increasing the expression of the chlorophyll biosynthetic gene leads to increased pigment synthesis^47^. In addition, AMF stimulates the production of secondary metabolites, including carotenoids and phenolic compounds^48^. AMF also increased tolerance in plants by regulating proline accumulation, and the mycorrhizal plants accumulated higher root and leaf proline contents than non-mycorrhizal plants^49^. Moreover, proline is considered an essential regulator that enhances plant resistance to various biotic stresses^50^.

## Conclusion

The results of the current study revealed that the lupine plant responded by varying degrees to different species of mycorrhizae. Of the four evaluated arbuscular mycorrhizal fungi (AMF) species, *Entrophospora lutea*, and *R. intraradices* exhibited a promising association with lupine plants by reducing damping-off and root-rot diseases in comparison with the chemical fungicide Rizolex-T as well as enhancing the growth and yield. Although Rizolex-T and AMF were equally effective, AMF offers eco-friendly and sustainable alternatives. These results emphasize the potential of these specific AMF species for future applications to improve the growth and productivity of lupine.

## Methods

### Lupine cultivar

Lupine seeds of cv. Giza 2 were obtained from the Legume Res. Dept., Field Crops Res. Inst., ARC, Giza, Egypt.

### Pathogen

#### Isolation and morphological identification

*Rhizoctonia solani* Kühn was obtained from lupine plants naturally infected and exhibiting damping-off and root rot symptoms in Sakhaa, Kafr El-Sheikh Governorate. The fungus was identified and its pathogenicity confirmed based on cultural and microscopic features^51,52^. Inoculum preparation was carried out according to the method outlined by Atwa et al.^53^.

#### Molecular identification

Extract of genomic DNA from pure fungal cultures were done using the CTAB (cetyltrimethylammonium bromide) method^54^. The internal transcribed spacer (ITS) region of ribosomal DNA was amplified using universal primers ITS1 (5′-TCCGTAGGTGAACCTGCG G-3′) as forward and ITS4 (5′ TCCTCCGCTTATTGA TATGC-3′) as reverse^55^. PCR amplification products were separated on a 1.5% agarose gel and visualized under UV light. The resulting amplicons were purified and sequenced. Sequences were compared to those in the NCBI (National Center for Biotechnology Information) GenBank database using CLUSTAL W in MEGA11 software^56,57^. A phylogenetic tree was constructed using the Jukes–Cantor model^58^. The analysis confirmed the identity and genetic relationship of the isolate with known *R. solani* strains.

#### AMF species and inoculum preparation

Four species of AMF were obtained from the laboratory of the Mycology and Plant Dis. Survey Dept, ARC, Giza, Egypt (Table 7), and were previously identified using fatty acid methyl ester profiles^59^. The wet sieves technique was used to collect and isolate the AFM spores^60^. The AM fungi were grown for three months in a multispore pot culture containing a 2:1:1:1 (w/w) mixture of autoclaved Holland peat moss, vermiculite, clay, and sand, with Sudan grass serving as the host plant. After being sieved through a 500-µm mesh, the inoculum was combined with 1% methylcellulose as a coating material^61^. The microbial inoculum was applied at a rate of around 300 spores/gram to ensure that the lupine seed surface had at least 30 spores^62^. The mixed treatment included equal amounts of all four AMF species.

**Table 7:** List of arbuscular mycorrhizal fungi (AMF).

Isolate no.	Isolate name	Sequenced number	Former name
1	*Entrophospora etunicata*	MT012422	*Glomus etunicatum*
2	*Rhizophagus clarus*	MW421738.1	*Glomus clarus*
3	*Rhizophagus intraradices*	MW410779	*Glomus intraradices*
4	*Entrophospora lutea*	PP069247	*Glomus luteum*

#### Root-nodule bacteria treatment

A formulation of *Rhizobium* bacteria (*Bradyrhizobium* sp. Lupinus) was obtained from the Biofertilizers Production Unit, Soils, Water and Environment Research Institute (SWERI), (ARC), Giza, Egypt. It was applied at a rate of 5 g per pot at sowing. However, about 50 kg of moistened fine sandy soil was mixed with 800 g of rhizobium formulation per feddan and incorporated into the seed furrow during sowing.

#### Fungicidal treatment

Lupine seeds were treated with Rizolex-T 50% WP (containing 20% Tolclophos-methyl and 30% Thiram), supplied by Sumitomo Chemical Company Ltd., at the recommended rate of 3 g/kg of seed, using a 1% methylcellulose solution as an adhesive.

### Greenhouse experiments

#### Seed preparation

Lupine seeds were disinfected with 1% sodium-hypochlorite solution for 2 min, finally washed with sterile distilled water, and air dried.

#### Pot preparation and cultivation

Thirty-centimeter diameter pots were sterilized using a 5% formalin solution and filled with steam-disinfected sandy clay soil (1:2 v/v) as outlined by Atwa^63^ outlined. The experiment was conducted at the Plant Pathology Research Institute, ARC, Giza, Egypt. The pots were arranged in a completely randomized design (CRD), with twelve pots assigned to each treatment as follows: (1) *Entrophospora etunicata*; (2) *Rhizophagus clarus*; (3) *Rhizophagus intraradices*; (4) *Entrophospora lutea*; (5) Mixture; (6) Rizolex-T; (7) and (8) seeds coated with peat moss and vermiculite-based formulation, serving as untreated control for both infested and non-infested soil.

#### Plant growth assessment

After 60 days, twelve plants (four replicates) were uprooted. Roots were washed using slow-running water to remove soil particles and organic debris. Then, the number of nodules per root system was counted after detaching from main and secondary (lateral) roots. Shoots were cut at the soil line to measure the length. Shoots, roots, and nodules were placed in paper bags and oven-dried at 70 °C for 48 h, then weighed, and the averages were recorded. Macro- and micro-elements were estimated in the shoots. The dry plant samples were ground and prepared for wet digestion using H_2_SO_4_ and H_2_O_2_ methods as described by Page et al.,^64^. The digests were then subjected to the measurement of macronutrients^65^ and micronutrients^56^. Measurement of nitrogenase activity was recorded as described by Hardy et al.,^67^.

#### Mycorrhizae colonization of lupine root

The percentages of root colonization by mycorrhizal isolates were determined 60 days after sowing. The root’s staining technique was applied according to^68^. Root colonization levels were estimated on 5 groups of 10 root segments (1 cm long) randomly chosen and examined under the light microscope for the presence of fungal structures. Root colonization percentage was calculated according to Phillips and Hayman^60^:

$$\text{Root colonization} \% \:=\:\:\:\frac{\:\text{N}\text{o}.\:\text{o}\text{f}\:\text{c}\text{o}\text{l}\text{o}\text{n}\text{i}\text{z}\text{e}\text{d}\:\text{r}\text{o}\text{o}\text{t}\:\text{f}\text{r}\text{a}\text{g}\text{m}\text{e}\text{n}\text{t}\text{s}\:}{\text{N}\text{o}.\:\text{o}\text{f}\:\text{t}\text{o}\text{t}\text{a}\text{l}\:\text{r}\text{o}\text{o}\text{t}\:\text{f}\text{r}\text{a}\text{g}\text{m}\text{e}\text{n}\text{t}\text{s}}\: \times 100$$

#### Disease incidence and disease severity

The disease incidence (DI) of pre- and post-emergence damping-off was recorded 15 and 30 days after planting. The survival plants were recorded after 45 days under greenhouse and field conditions, as described by Atwa^63^.

The percentage of reduction or increase relative to the infected control was calculated as follows:

$$\text{Reduction or increasing} \% \:=\:\:\:\frac{\text{D}\text{i}\text{s}\text{e}\text{a}\text{s}\text{e}\:\text{i}\text{n}\text{c}\text{i}\text{d}\text{e}\text{n}\text{c}\text{e}\:\left(\text{D}\text{I}\right)\:\text{o}\text{f}\:\text{i}\text{n}\text{f}\text{e}\text{c}\text{t}\text{e}\text{d}\:\text{c}\text{o}\text{n}\text{t}\text{r}\text{o}\text{l}\:-\:\text{D}\text{I}\:\text{o}\text{f}\:\text{t}\text{r}\text{e}\text{a}\text{t}\text{m}\text{e}\text{n}\text{t}\:\:}{\text{D}\text{I}\:\text{o}\text{f}\:\text{i}\text{n}\text{f}\text{e}\text{c}\text{t}\text{e}\text{d}\:\text{c}\text{o}\text{n}\text{t}\text{r}\text{o}\text{l}}\: \times 100\:$$

A numerical rating scale (0–5) was used to rate the plants for disease severity^69^, and the following formula was used to determine the disease index of root rot:

$$\text{Disease Index} \:=\:\:\frac{\:{\Sigma\:}\:\left(\:\text{n}\text{u}\text{m}\text{b}\text{e}\text{r}\:\text{o}\text{f}\:\text{r}\text{o}\text{o}\text{t}\text{s}\:\text{t}\text{e}\text{s}\text{t}\text{e}\text{d}\:\text{i}\text{n}\:\text{e}\text{a}\text{c}\text{h}\:\text{g}\text{r}\text{a}\text{d}\text{e}\:x\:\:\text{d}\text{e}\text{g}\text{r}\text{e}\text{e}\:\text{o}\text{f}\:\text{d}\text{a}\text{m}\text{a}\text{g}\text{e}\:\right[0-5\left]\right)}{\text{t}\text{o}\text{t}\text{a}\text{l}\:\text{n}\text{u}\text{m}\text{b}\text{e}\text{r}\:\text{o}\text{f}\:\text{t}\text{e}\text{s}\text{t}\text{e}\text{d}\:\text{p}\text{l}\text{a}\text{n}\text{t}\text{s}\:x\:\text{t}\text{h}\text{e}\:\text{h}\text{i}\text{g}\text{h}\text{e}\text{s}\text{t}\:\text{d}\text{e}\text{g}\text{r}\text{e}\text{e}\:\text{o}\text{f}\:\text{i}\text{n}\text{f}\text{e}\text{c}\text{t}\text{i}\text{o}\text{n}\:\left(5\right)}\:\:x\:100$$

#### Field experiments

In a field known to have a history of root rot at Giza Agricultural Research Station, ARC, Egypt, experiments were carried out using a randomized block design (RBD) during the 2021 and 2022 seasons at the beginning of November. The treatments were as follows: (1) *Entrophospora etunicata*; (2) *Rhizophagus clarus*; (3) *Rhizophagus intraradices*; (4) *Entrophospora lutea*; (5) Mixture; (6) Rizolex-T; and (7) untreated control, with four replicates for each treatment (28 plots). The plot was 10.5 m^2^ (3.5 m length × 0.6 m width) with five rows. Two lupine seeds were sown at a row spacing of 20 cm apart on either side of the row ridge. The other required agricultural methods, such as irrigation and fertilization, were followed according to the recommendations of the Egyptian Ministry of Agriculture. During harvest, ten lupine plants were taken at random from the inner rows of each plot to measure the growth parameters and estimate yield (ton/feddan).

#### Enzyme activity assay

Plant tissue (4 g) from each treatment (15 days after sowing) was homogenized at 0 °C in 6 ml of 0.1 M phosphate buffer (pH 7.0) with a small amount of neutral sand. The homogenate was filtered, centrifuged at 3000 rpm for 15 min at 4 °C, and the resulting supernatant (crude enzyme extract) was either stored at − 20 °C or used immediately for the assay.

#### Assay of peroxidase (PO)

Peroxidase (PO) activity was determined according to Chakraborty & Chatterjee^70^. The reaction mixture consisted of 1.5 ml enzyme extract and 5 ml freshly prepared pyrogallol reagent, and the absorbance was adjusted to zero before initiating the reaction with 0.5 ml of 1% H_2_O_2_. Peroxidase activity was determined spectrophotometrically as the change in absorbance per minute at 430 nm.

#### Assay of polyphenol oxidase (PPO)

Polyphenol oxidase enzyme (PPO) was determined according to Sadasivam and Manickam^71^. For activity measurement, 2 ml of enzyme extract was mixed with 3 ml of phosphate buffer in a cuvette and adjusted to zero absorbance at 495 nm. The reaction was initiated by adding 1 ml of 0.01 M catechol in phosphate buffer (0.4 mg/ml), and PPO activity was recorded as the change in absorbance per minute at 495 nm.

#### Determination of phenolic contents

Phenolic compounds were extracted 15 days after sowing according to Sutha et al.^72^. Fresh plant tissue (5 g) from each treatment was homogenized in 30 ml of 80% ethanol and incubated at 50 °C for 30 min with shaking, followed by centrifugation at 10,000 rpm for 10 min. The pellet was re-extracted twice, and pooled supernatants were washed with petroleum ether to remove chlorophyll. The alcohol fraction was evaporated under vacuum at 45 °C, and the residue was dissolved in isopropanol and stored at − 20 °C. Total phenolic content was determined according to Snell and Snell^73^ by mixing 0.5 ml of the extract with 0.25 ml HCl, boiling in a water bath for 10 min, cooling, then adding 1 ml Folin–Ciocalteu reagent and 6 ml of 20% Na_2_CO_3_, and diluting to 10 ml with distilled water (30–35 °C). After incubation in the dark for 30 min, absorbance was measured at 520 nm.

#### Determination of proline content

Approximately 0.5 g of fresh leaves (40 days after sowing) were ground into powder with liquid nitrogen and extracted in 3% sulfosalicylic acid. After centrifugation at 8000 rpm for 10 min, the supernatant (2 mL) was mixed with 2 mL of reagent (2 mL ninhydrin, 2 mL glacial acetic acid) and incubated at 100 °C for 40 min. The reaction was then terminated in an ice bath. The reaction mixture was extracted with 4 mL of toluene, and the absorbance was measured at 520 nm^74^.

#### Determination of photosynthetic pigments (Chlorophyll a, b, and carotenoid content)

The content of photosynthetic pigments was determined according to the method of Arnon^75^. A 0.5 g sample of fresh leaves was crushed with 80% acetone and centrifuged at 10,000 rpm for 5 min at 4 °C. From the supernatant, 1mL was used to measure the concentrations of chlorophyll at 663 nm, chlorophyll b at 645 nm, total chlorophyll, and carotenoids at 480 nm.

### Statistical analysis

The data obtained were subjected to computer statistical software (ASSISTAT) originated by Silva & Azevedo’s^76^. Data analyzed using analysis of variance (ANOVA), and mean values were compared using Duncan’s multiple range test at a significance level of *P* ≤ 0.05.

## Data Availability

All data generated or analyzed during this study are included in this published article.
